# Safety and function of programmable ventriculo-peritoneal shunt valves: An in vitro 7 Tesla magnetic resonance imaging study

**DOI:** 10.1371/journal.pone.0292666

**Published:** 2023-10-11

**Authors:** Bixia Chen, Philipp Dammann, Ramazan Jabbarli, Ulrich Sure, Harald H. Quick, Oliver Kraff, Karsten H. Wrede

**Affiliations:** 1 Erwin L. Hahn Institute for Magnetic Resonance Imaging, University Duisburg-Essen, Essen, North Rhine Westphalia, Germany; 2 Department of Neurosurgery, University Hospital Essen, University Duisburg-Essen, Essen, North Rhine Westphalia, Germany; 3 High-Field and Hybrid MR Imaging, University Hospital Essen, University Duisburg-Essen, Essen, North Rhine Westphalia, Germany; Linköping University, SWEDEN

## Abstract

**Objective:**

The quantity of ultra-high field MRI neuroimaging studies has rapidly increased. This study tests function, safety, and image artifacts of two frequently implanted programmable ventriculo-peritoneal (VP) shunt valves in a 7T MRI system.

**Methods:**

All tests were performed using a whole-body 7T MRI system. Three proGAV 2.0 and 3 CODMAN CERTAS® Plus programmable VP-shunt valves were tested in three steps. 1) Deflection angle tests close to the bore opening at the location of a static magnetic field gradient of 3–5 T/m. 2) Valves were fixed on a spherical phantom in 3 positions (a. lateral, b. cranial, c. cranial with 22.5° tilt anteriorly) and assessed for keeping the programmed pressure setting and reprogrammability. 3) Valves were fixed on the phantom and positioned lateral in a radiofrequency head coil. MRI scans were performed for both models, including MPRAGE, GRE and SE sequences.

**Results:**

Deflection angles were moderate (13°, 14°, 13°) for the proGAV valves and close to critical (43°, 43°, 41°) for the CODMAN valves at the test location. Taking a scaling factor of 2–3 for the maximum spatial magnetic field gradient accessible to a patient within the magnet bore into account renders both valves MR unsafe regarding ferromagnetic attraction. The proGAV valves kept the pressure settings in all positions and were reprogrammable in positions a. and b. In position c., reprogrammability was lost. The CODMAN valves changed their pressure setting and reprogrammability was lost in all positions. MR image signal homogeneity was unaltered in the phantom center, artifacts limit the assessability of structures in close vicinity to the valves.

**Conclusion:**

Both tested programmable VP-shunt valves are MR unsafe for 7T systems. Novel programming mechanisms using permanent magnets with sufficient magnetic coercivity or magnet-free mechanisms may allow the development of programmable VP-shunt valves that are conditional for 7T MR systems.

## Introduction

In the last years, the quantity of ultra-high-field (UHF) magnetic resonance imaging (MRI) studies demonstrating diagnostic benefits in neuroimaging has increased rapidly [1–3]. The first 7 Tesla (T) MRI system received clearance for clinical use in 2017 [4].

Today, MRI is the primary diagnostic tool in neurosurgical patients and mandatory for follow-up of most patients after surgical treatment. After cranial fixation plates [5], the most commonly used neurosurgical implants are programmable ventriculo-peritoneal (VP) shunts. They are used for hydrocephalus treatment and drain excess cerebrospinal fluid to the intraperitoneal space. In modern adjustable VP shunts, an adjustable magnetic pressure regulating mechanism allows for non-invasive adjustment and regulation of the CSF flow rate after implantation. They are known to resist unwanted valve pressure changes when exposed to MRI scanners with field strengths up to 3 Tesla [6].

Intracranial pathologies leading to hydrocephalus with an indication for VP shunt implantation are various and affect patients of all ages [7]. Common underlying conditions for shunt dependency include congenital malformations [8], hemorrhagic events [9], traumatic brain injury [10], vascular pathologies [11], tumors of the nervous systems [12, 13], and infectious diseases [14].

This in vitro study primarily assesses the function, but also includes indications for safety, and image artifacts of two worldwide frequently implanted programmable VP-shunt valves in a 7T whole-body MRI system.

## Materials and methods

### Ethics statement

Approval by the local university institutional review board was not necessary for this in-vitro safety study.

### Scanner and coil systems

Tests were performed using a whole-body MRI system (MAGNETOM 7T; Siemens Healthcare GmbH, Erlangen, Germany) equipped with a 1-channel transmit/32-channel receive head radiofrequency coil (Nova Medical, Wilmington, Massachusetts, USA). The 7T magnet of the system is passively shielded.

### Shunt valve systems

**Fig 1:**
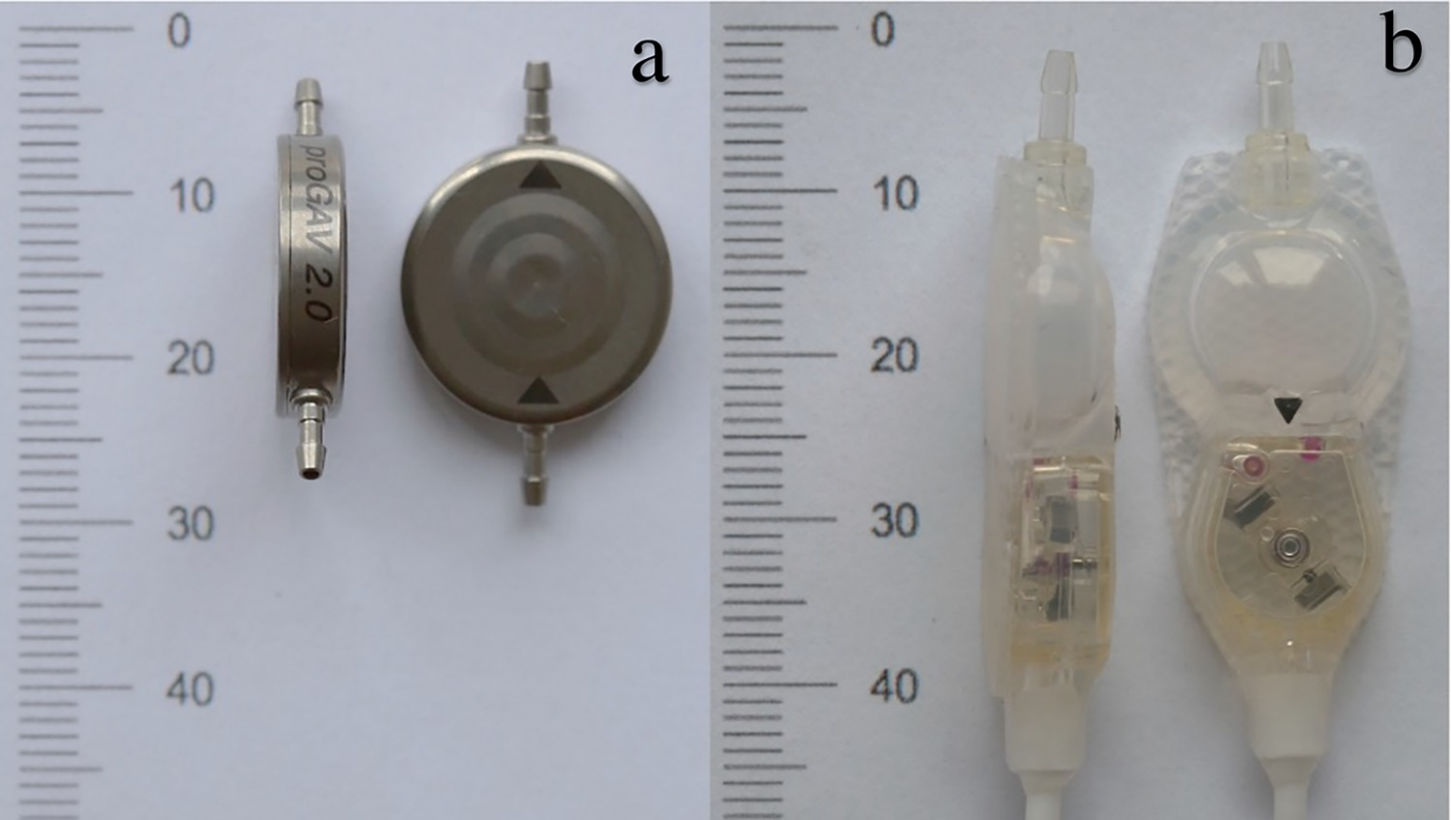
Evaluated programmable shunt systems. a: Frontal and lateral view of the Miethke proGAV 2.0 programmable shunt valve. b: Frontal and lateral view of the Codman CERTAS Plus programmable shunt valve.

The two most frequently implanted programmable shunt valves (Fig 1) were chosen for the test. The first model was the proGAV 2.0 programmable VP-shunt valve (Christoph Miethke GmbH, Potsdam, Germany) which consists of an adjustable pressure unit integrated in a titanium body. Opening pressure levels can be selected between 0 mm H_2_O and 200 mm H_2_O.

The second model was the Codman CERTAS® Plus programmable VP-shunt valve (Codman & Shurtleff, Inc., Raynham, Massachusetts, USA), a valve with an adjustable pressure unit in a silicone body. Pressure levels can be set between 25 mm H_2_0 and 215 mm H_2_0.

Both devices are adjustable through the skin with a magnetic programming tool and have previously been tested to be 3 T MR conditional [15, 16]. Three valves of each type were tested in a three-step procedure in this study.

### Deflection angle test

**Fig 2:**
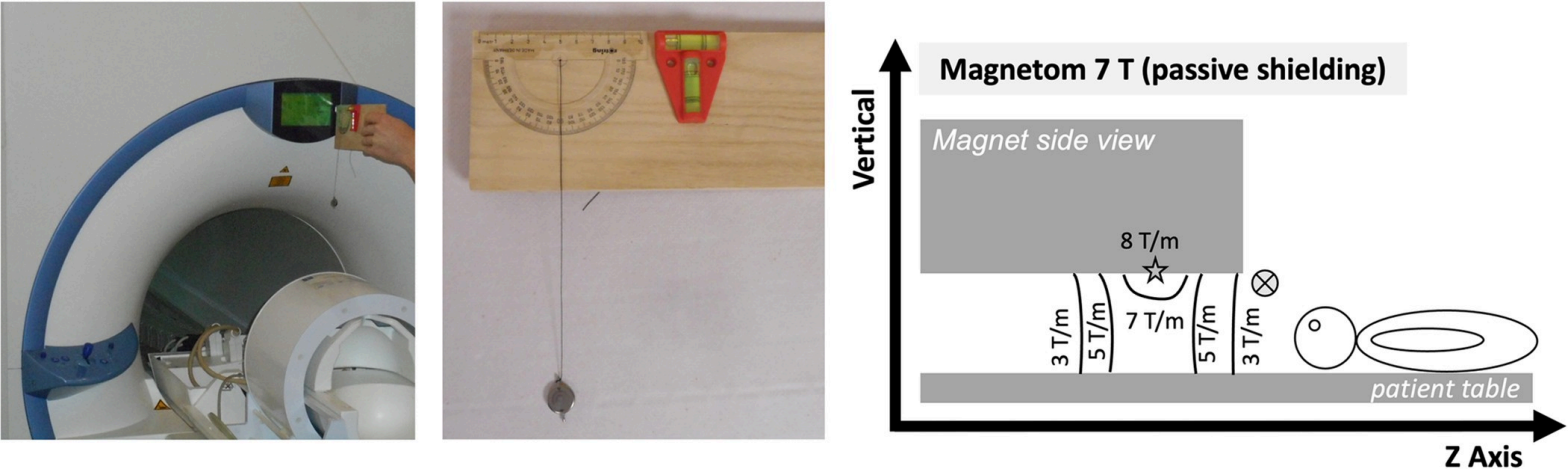
Deflection angle test.

Deflection angle tests on the basis of standard ASTM F2052 [17] were performed close to the bore opening of the 7T MRI system at the location of a static magnetic field gradient of 3–5 T/m, as taken from the MR compatibility data sheet manual provided by the MR system vendor. Fig 2 shows the test device and the positioning in front of the MR system. The test device consisted of a wooden plate with a mounted protector and a thin sewing thread to attach the valves. Tubular spirit levels helped to keep the hand-held device horizontal during measurements. A deflection angle of > 45° is considered as critical because then the forces due to ferromagnetic attraction on the implant are higher than the forces due to gravitation.

Exemplary image of the deflection angle test with a hand-held device that is shown in detail in the center. Side view of the 7T magnet (front quadrant, patient end) and its respective, exemplary distribution of the spatial, static magnetic field gradient. The encircled X marks the position of the deflection tests. The star marks the places where the force on a magnetically saturated ferromagnetic object is greatest. Note, that the deflection tests may provide an indication of safety rather than absolute values as the test procedure did not follow ASTM F2052 guidelines in detail nor have the spatial gradients been verified by additional measurements.

### Valve programming mechanism tests

**Fig 3:**
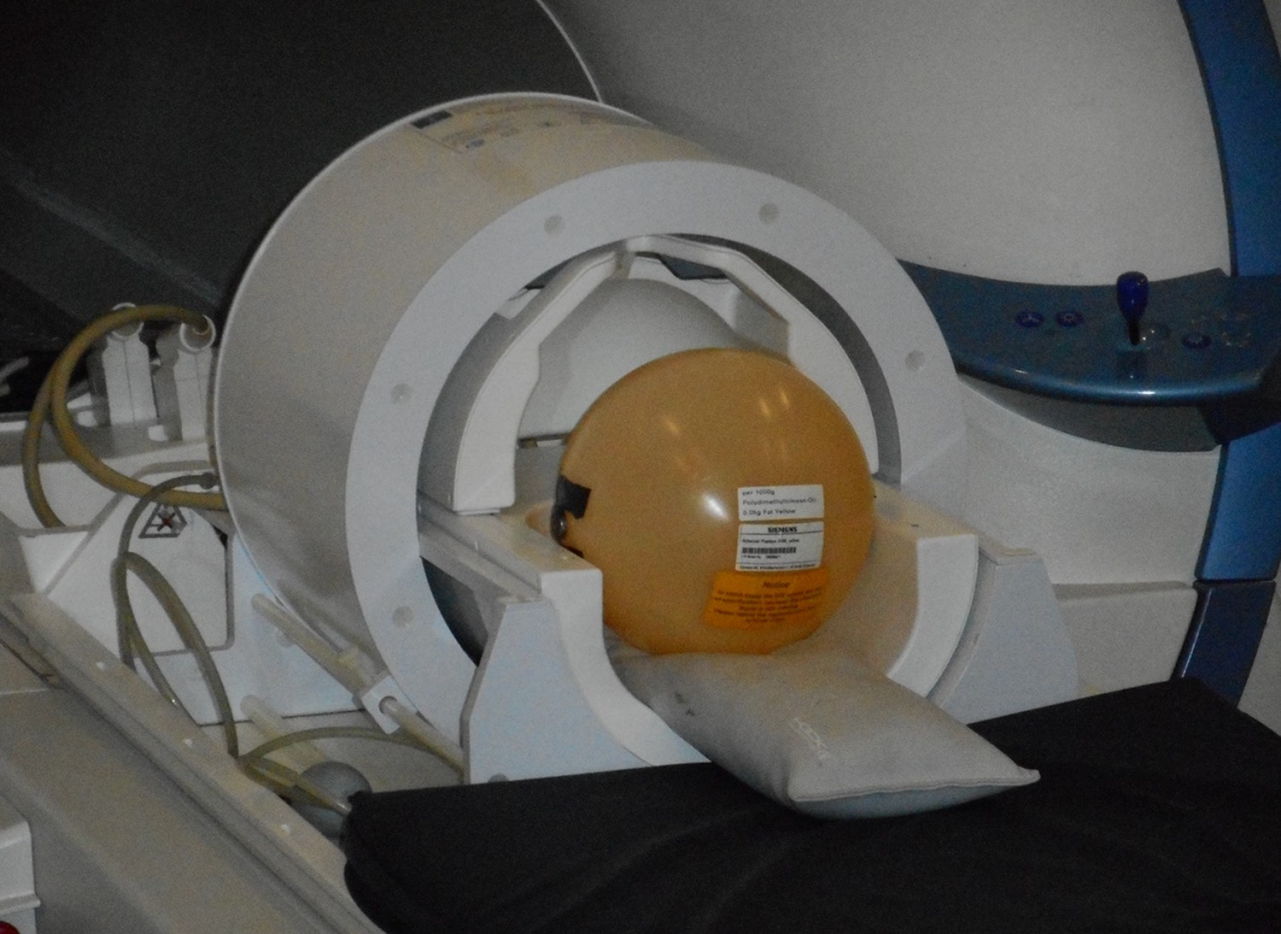
MRI measuring setup. An adjustable shunt valve is laterally fixed to the spherical phantom which is placed in a 1-channel transmit/32-channel RF head coil.

**Fig 4:**
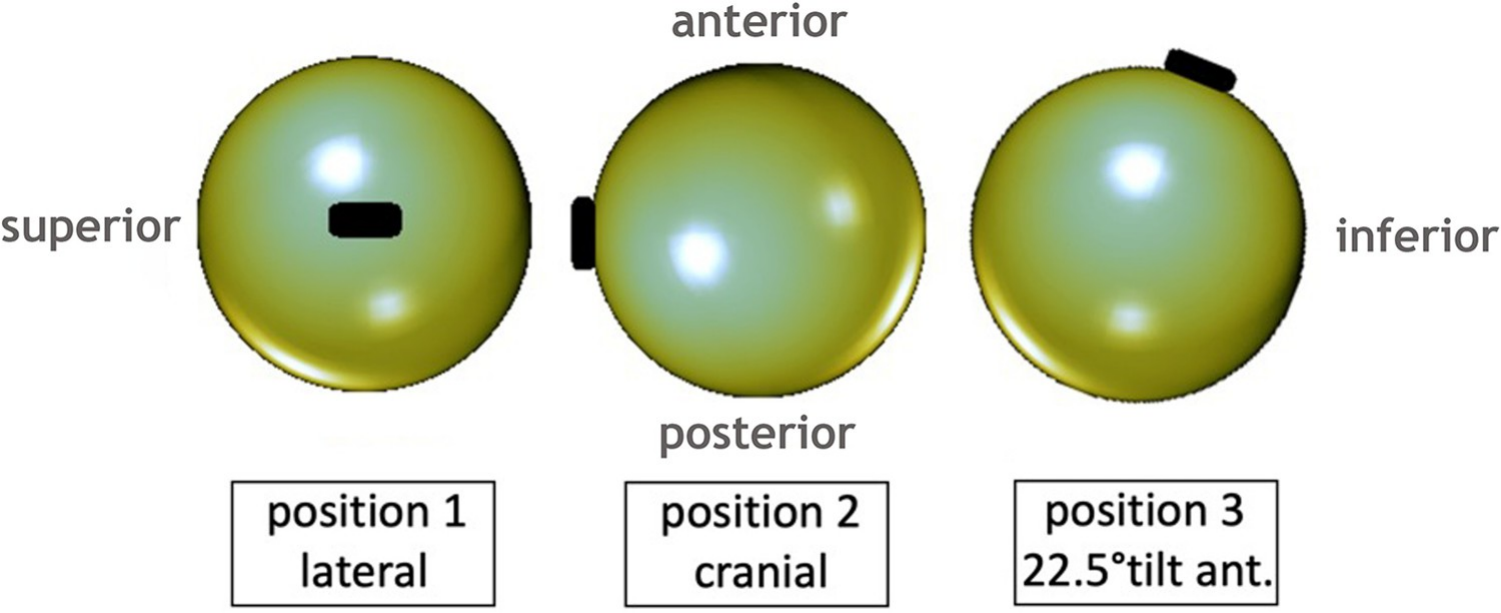
Shunt valve positions. Phantom positions shown from a lateral view. The shunt valve (depicted in black) is fixed to the spherical phantom strictly lateral (position 1), cranial (position 2), and 22.5° tilt anteriorly (position 3).

The valves were fixed on a standard spherical MRI phantom (Siemens, Erlangen, Germany) and tested for keeping the programmed pressure setting and ability to be reprogrammed. Positioning on the surface of the phantom corresponds to the position of an implanted VP shunt on the outer surface of the skull bone. The phantom with the valve was positioned in the radiofrequency (RF) head coil and centered in the magnet’s isocenter using a motorized patient table with a constant speed of 10 cm/s. Then the phantom was removed from the scanner, and the valve was tested for keeping the programmed pressure setting and reprogramming ability using vendor-specific tools. This procedure was repeated three times for the two shunt models. Three valves of each shunt model were placed in one of the following positions on the phantom: 1. strictly lateral; 2. strictly cranial, 3. cranial with 22.5° tilt anteriorly. Fig 3 illustrates the phantom placement in the 32-channel RF head coil, while Fig 4 shows the valve positions 1 to 3 from a lateral view of the phantom. A standard hiking compass (Suunto A-10, Finland) with a high grade steel needle in a liquid filled capsule was used to determine a possible repolarization of the magnetic valves after exposure to 7T.

### Image artifacts

A single sample valve was fixed on the standard spherical MRI phantom and positioned strictly lateral in the RF head coil. Scans were performed for both models separately. Additionally, the empty proGAV 2.0 titanium casing was examined. Before acquiring the diagnostic sequences, B_0_ shimming was performed manually in 2–3 iterations using the vendor-provided sequences. Acquired sequences included B_1_ mapping, magnetization-prepared rapid acquisition gradient-echo (MPRAGE) [18], spin echo (SE), and gradient echo (GRE) sequences according to ASTM F2119 [19]. Imaging protocols are shown in Table 1. Image evaluation was performed in consensus reading by two experienced raters (KHW and BC) using an open-source medical image viewer (Horos; http://www.horosproject.org/). We have evaluated the artifacts with 2D GRE and SE sequences in two orientations, i.e., coronal with HF phase encoding and transversal with RL phase encoding. In addition, a 3D MPRAGE sequence was used. Image homogeneity and artifacts caused by the shunt valves were measured and evaluated on a five-point likert scale (5 = excellent, without artifacts; 4 = good, minimal artifacts < 5 mm; 3 = moderate, intermediate artifacts 5.1–15 mm; 2 = poor, distinct artifacts 15.1–25 mm; 1 = non-diagnostic, strong artifacts > 25 mm) The maximum diameters of artifacts around the shunt valves were quantified for each imaging sequence. Complete image datasets are available via depository: https://doi.org/10.5281/zenodo.8318139.

**Table 1:** Imaging protocols.

Sequence	TR [ms]	TE [ms]	TA [min:sec]	Flip angle [°]	Matrix	Acquired voxel size [mm^3^]
**GRE**	200	15	1:42	30	256 x 256	0.7 x 0.7 x 2.0
**MPRAGE**	2500	1.54	6:13	6	384 x 384	0.7 x 0.7 x 0.7
**SE**	514	20	2:15	90	256 x 256	0.7 x 0.7 x 2.0

GRE: Gradient Echo

MPRAGE: Magnetization-Prepared Rapid Gradient-Echo

SE: Spin Echo

## Results

An overview of the results for assessment steps 1 to 3 are shown in Table 2.

**Table 2:** Deflection angles of programmable shunt valves at the location of the 3 T/m static magnetic field gradient, ability to keep the pressure level and reprogrammability.

Valve	Deflection angle	Pressure level kept			Reprogrammability		
		*0°*	*90°*	*45°*	*0°*	*90°*	*45°*
Codman Certas Plus no. 1	43°	No	No	No	No	No	No
Codman Certas Plus no. 2	43°	No	No	No	No	No	No
Codman Certas Plus no. 3	41°	No	No	No	No	No	No
Miethke proGAV 2.0 no. 1	13°	Yes	Yes	Yes	Yes	Yes	No
Miethke proGAV 2.0 no. 2	14°	Yes	Yes	Yes	Yes	Yes	No
Miethke proGAV 2.0 no. 3	13°	Yes	Yes	Yes	Yes	Yes	No

### Deflection tests

All valves deflection angles were moderate (13°, 14°, and 13°) for the proGAV 2.0 programmable VP-shunt valves and critical (43°, 43°, and 41°) for the CODMAN CERTAS® Plus programmable VP-shunt valves at the test location. According to the MR system compatibility data sheets of the first generation passively shielded MR system (MAGNETOM 7T) and of the third generation actively shielded MR system (MAGNETOM Terra) these values need to be multiplied by a factor of 2–3 to take into account the spatial magnetic field gradients accessible to the patient within the boreliner of the respective MRI system.

### Ability to keep programmed pressure setting and for reprogramming

**Fig 5:**
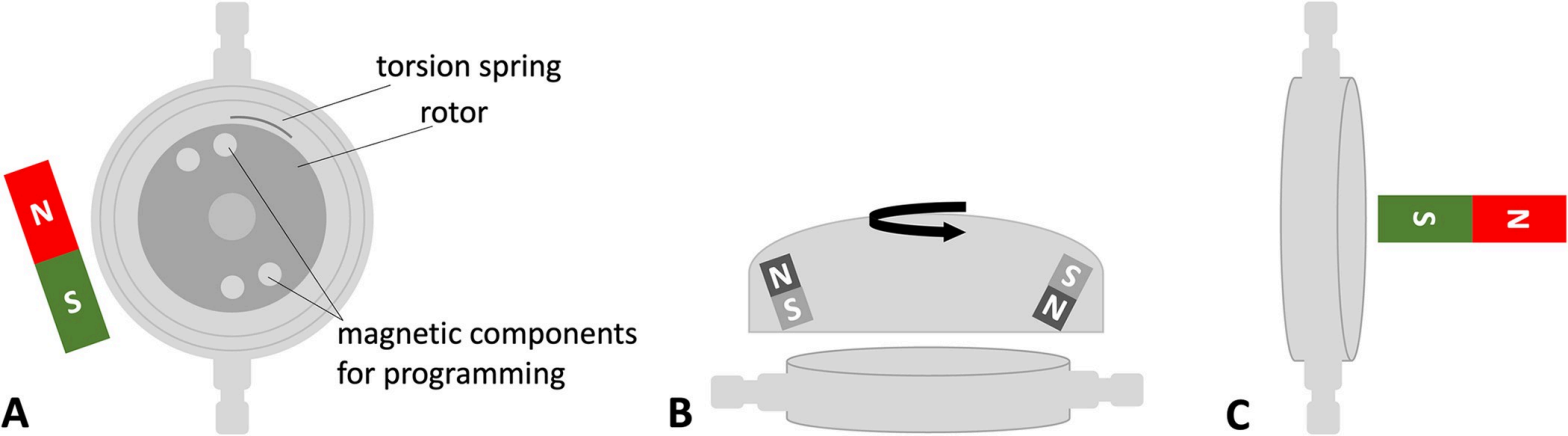
Polarization test. Schematic view of the adjustable pressure unit for the proGAV 2.0 valve with its magnetic field orientation for programming (A). In (B) the adjustment tool for programming is placed centrally above the valve. After exposure to 7T the programming functionality was lost due a 90 degree shifted magnetic field orientation within the rotor component (C) in comparison to the original setting (A).

The proGAV 2.0 programmable VP-shunt valves kept the programmed pressure settings with re-programmability in positions 1 and 2 on the phantom. The valves kept their pressure setting, but the re-programmability was lost after the first test with position 3 on the phantom. The compass showed a repolarization of the magnetic components that are the crucial part of the programming mechanism within the valves. After the third exposure to 7T the direction of the magnetic field within the adjustable pressure unit changed from an in-plane orientation towards both catheter connectors to a 90 degree shifted orientation pointing upside-down at the housing (Fig 5). Videos of the tests are available in the supplementary material. The CODMAN CERTAS® Plus programmable VP-shunt valves changed their pressure setting and reprogrammability was lost after the first test in position 1, 2 and 3 on the phantom, respectively.

### Imaging artifacts

**Fig 6:**
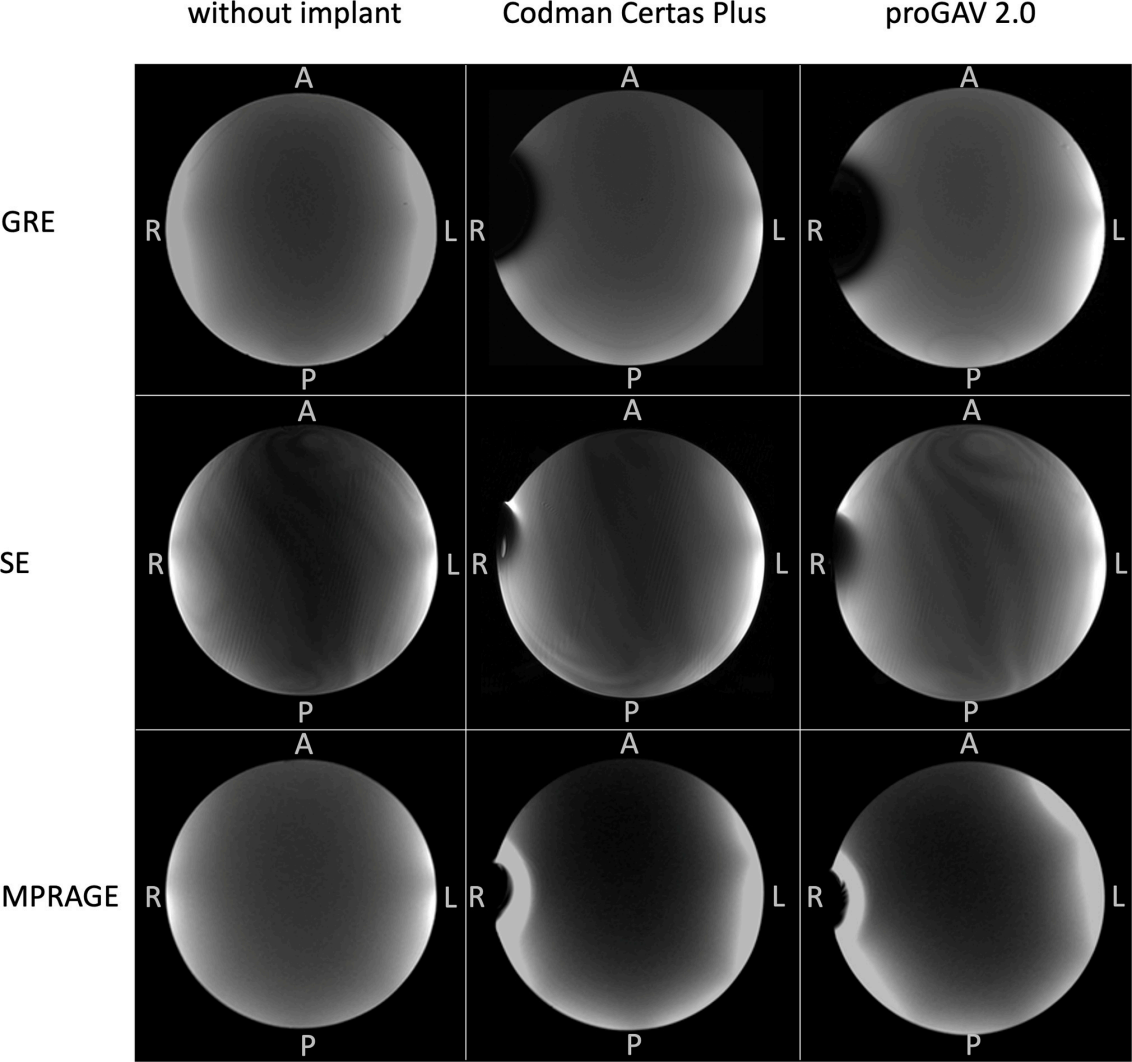
Image artifacts. Gradient echo (GE), spin echo (SE) and Magnetization-Prepared Rapid Gradient-Echo (MPRAGE) transversal sequences of the spherical phantom only, the phantom with a Codman CERTAS Plus valve attached, and the phantom with a proGAV 2.0 valve attached. Image artifacts of the Codman valve reached up to 24mm in the GRE sequence, 12 mm SE, and 20 mm in the MPRAGE sequence. ProGAV valve artifacts had an extend up to 28 mm in the GRE, 12 mm in the SE, and 21 mm in the MPRAGE sequence.

The image signal homogeneity was unaltered in the center of the phantom. Image artifacts adjacent to the shunt valves were tolerable. Assessment results are shown in Table 3. Fig 6 illustrates image homogeneity and artifacts for both valve systems in different MR sequences. Artifact extensions reached up to 28 mm. Artifacts caused by the proGAV 2.0 titanium casing without a magnet led to approximately 2-fold smaller artifacts than with a valve mechanism.

**Table 3:** Assessment of imaging quality evaluated on a 5-point-scale.

		MPRAGE	GRE	SE
**Codman Certas Plus**	*Artifact assessment*	2	2	3
	*Max*. *artifact diameter*	20 mm	24 mm	12 mm
**Miethke proGAV 2.0**	*Artifact assessment*	2	1	3
	*Max*. *artifact diameter*	21 mm	28 mm	12 mm

(5 = excellent, without artifacts; 4 = good, minimal artifacts < 5 mm; 3 = moderate, intermediate artifacts 5.1–15 mm; 2 = poor, distinct artifacts 15.1–25 mm; 1 = non-diagnostic, strong artifacts > 25 mm).

GRE: Gradient Echo

MPRAGE: Magnetization-Prepared Rapid Gradient-Echo

SE: Spin Echo

## Discussion

Several commonly used cranial implants have been tested for MR safety in 7T MRI systems [20–22], among others, cranial fixation plates [5, 23] and shunt assistants [24]. The proGAV 2.0 programmable VP-shunt valves showed a moderate deflection in the static magnetic field of the scanner that can be considered MR conditional for a neurosurgical implant. The nearly critical deflection of the CODMAN CERTAS® Plus programmable VP-shunt valves was probably caused by the larger permanent magnets used in the programming mechanism, and they can only be considered marginally conditional for a neurosurgical implant. After the programming mechanisms were dysfunctional, the deflection angles of the proGAV 2.0 valves increased to a critical level (43°, 38°, and 44°) which might be due to remanence effects of the material during MRI measurements. The deflection angles of the Codman CERTAS Plus valves stayed almost constant (39°, 36°, and 38°). Both programmable shunt valves should be treated with caution for exposure to a 7T MRI system. Note that the deflection tests were performed on the first generation’s passively shielded 7T magnet. For the third-generation actively shielded magnets (MAGNETOM Terra, Siemens Healthcare GmbH) the maximum spatial magnetic field gradient accessible to the patient within the bore increases to 7–10 T/m [22]. Taking this scaling into account, the deflection angle will be above 45° and hence, rendering the VP-shunts MR unsafe. However, since the deflection tests were performed with a hand-held device and without verification of the spatial gradients the presented results should be treated as indications for safety rather than absolute values. In addition, all shunt valves lost their re-programmability after a series of exposures to the static magnetic field of the scanner. The insufficient magnetic coercivity of the permanent magnets used in the programming mechanisms renders the valves unusable after exposure to the static magnetic field of a 7 Tesla whole-body MRI scanner. Programming is performed with an adjustment tool that has two magnets on each side with opposite orientation, which allows rotor rotation within the valve’s main plain. If the magnetic domains within the rotor’s components are reorganized to a 90 degree shifted orientation due to the strong external magnetic field, programming functionality will be lost. Permanent magnets with higher magnetic coercivity in the shunts might help to overcome this issue. Alternatively, developing pressure-setting mechanisms completely without magnetic components could warrant the usability of shunt valves during UHF MRI scans. In principle, the examination routine for implant safety also includes the assessment of potential RF-induced heating. However, this step was not performed in this study because the deflection angles were already critical and the function of the pressure adjustment mechanism was lost after exposure to the magnetic field. The shunt valves unaltered the image signal homogeneity, and image artifacts adjacent to the valves were tolerable. The diagnostic assessability of structures located in close proximity to the valve may be limited.

## Conclusion

Both tested programmable VP-shunt valves are considered MR unsafe for use in 7T whole body MRI systems in their current form. Novel programming mechanisms with permanent magnets of sufficient magnetic coercivity or non-magnetic setting mechanisms may allow development of programmable VP-shunt valves that are conditionally safe for use in 7T whole body MRI systems.

## Supporting information

S1 File Magnetization test in an intact Miethke proGAV 2.0. In an intact Miethke proGAV 2.0 shunt valve, the compass shows a magnetic field within the adjustable pressure unit in an in-plane orientation towards both catheter connectors.
(MP4)

S2 File Magnetization test in a Miethke proGAV 2.0 after exposition to 7 Tesla MRI. After exposition to the 7 Tesla MRI, the compass shows a 90 degree shifted magnetic field orientation pointing upside-down at the housing of the Miethke proGAV 2.0 shunt valve.
(MP4)

## Abbreviations

GRE: Gradient echo
MPRAGE: Magnetization-prepared rapid acquisition gradient-echo
MRI: Magnetic resonance imaging
RF: Radiofrequency
SE: Spin echo
T: Tesla
UHF: Ultra-high-field
VP: Ventriculo-peritoneal
